# Tonga

**Published:** 1881-12

**Authors:** 


### TONGA.

A legel contest is now being waged between two leading firms of manufacturing pharmacists concerning the right to use this term. Strictly speaking, there can be but one side to this question, for if the right exists to copyright the name of any single newly discovered remedy there is an equal right to copyright all, and how much of a clog to medical progress this would be scarcely needs mention. One of these firms, be its motives what they may, is certainly right in denying the claim of anyone to copyright what is now generally accepted as a scientific term. The term in dispute is by no means a new one; it was in use fifty years ago, according to the Pharmaceutisches Centralhalle. How any one can claim copyright of it under such circumstances will puzzle an impartial observer. The medical profession is interested very deeply in this question, for it involves the right not to a specific preparation or mode of manufacture, but to the use of a single article of the materia medica, and if this claim of copyright be conceded the profession will suffer a series of extortions at the hands of unscrupulous pharmaceutical corporations.—*Chicago Med. Review.*

